# Fostering self-regulated learning in preschool through dynamic assessment methodologies

**DOI:** 10.1371/journal.pone.0298759

**Published:** 2024-03-21

**Authors:** Janete Silva Moreira, Paula Costa Ferreira, Ana Margarida Veiga Simão

**Affiliations:** CICPSI, Faculty of Psychology, University of Lisbon, Lisbon, Portugal; University of Oviedo, SPAIN

## Abstract

Self-regulated learning is a transversal competency which plays a central role in acquiring autonomy. This investigation aimed to support approaches that foster self-regulated learning in preschool. We proposed to improve preschoolers’ self-regulated learning strategies (i.e., forethought, performance, and self-reflection) through the educational intervention *Pipo and Mia*, *the magic knights*, hypothesizing different results when comparing levels of the program intervention. Participants included 115 preschoolers and their nine teachers. Teachers implemented the program to children, and engaged in professional training simultaneously. Aiming to validate the Dynamic Assessment of Self-regulation in Preschool (DASP) method socially, it was used as children’s pre and post-measure, and focus groups were conducted with teachers to assess its validity. Results showed improvements in children’s use of strategies, and some significant differences between intervention levels. Teachers highlighted the DASP method potentialities. The study’s contributions and constraints are discussed considering implications for practice, research, theory, and policy.

## Introduction

### Promoting self-regulated learning in preschool

Self-regulated learning (SRL) development in preschool is a relevant research field and some work has been devoted to the topic [1–3]. However, more investigation is needed considering children’s specificities, and the current social and educational challenges, underlying the need for policy makers, program leaders and professional development providers to ensure resources that support teachers and, ultimately, help students be more active and autonomous in their learning process [4, 5].

Preschool age offers developmental potentialities that play a unique opportunity in the SRL area [6]. Additionally, as preschool allows a certain flexibility on how learning contents are approached [7–9], this stage of life may be considered essential to promote SRL strategies. In school, children can benefit from opportunities provided by teachers’ daily practices and by structured educational interventions to train SRL strategies [10]. Some of the practices more commonly used by teachers to promote preschool quality and, consequently, stimulate children’s SRL and autonomy are time for planning, specific instructional content, group dialogue, problem-solving, and challenges [11, 12]. Moreover, children are frequently allowed to make choices and they can make simple decisions requiring strategic thinking about daily activities and materials, which is a direct manner to exercise their self-regulated competencies [6, 13, 14]. Recent educational interventions are intending to promote SRL strategies in preschoolers and, although the approaches are promising to increase the use of strategies, the results are not always satisfactory [1]. Furthermore, age-appropriate methodologies and instruments are needed [15], since those which are being used are not preschool curriculum-based neither are assessed in terms of social practice and sustainability to be applied beyond the research context.

Considering the arguments presented, the first aim of this study was to investigate whether preschoolers’ use of SRL strategies could be fostered after experiencing an educational intervention that uses authentic preschool activities and is delivered by the children’s preschool teachers.

### Interventions based on self-regulated learning: A support for educational resources and training

One of the goals of educational practices is to promote the learners’ active role, giving them the chance to train skills and reflect on their performance through problem solving or task resolution [11]. In preschool, although teachers organize activities where children can exercise their freedom of choice, the interventions where the activities are structured according to the SRL concept can achieve more evident results because the content validity is improved [16]. Also, SRL processes and strategies can be observed through children’s daily activities, potentiating the development of these skills in real and ecologic contexts [17]. The fact that SRL social cognitive approaches, specifically Zimmerman’s model [18], are defined as cyclic, multidimensional and dynamic, comparable to a continuous system reinforced by previous experience and open to new learning, provides a solid basis for educational resources and instruments. The model has three cyclic phases (i.e., forethought, performance, and self-reflection), the specific processes and strategies of each phase, and the dimensions associated to them (e.g., motivational, social, emotional, cognitive/metacognitive) can be differently explored to support children’s competencies [6]. Relying on the selection of the categories according to the SRL model [18] and the literature advice [16], the interventions have their content and predictive validity reinforced.

The tools and interventions that assess and promote young children’s SRL skills and present higher quality results are theoretically grounded in the SRL concept. They often use games [19], digital tools [20], and stories [21, 22] as central resources of the work. Relating to the latter, in preschool, the moment of “telling the story” is very appreciated, both by adults and children as they tend to identify with the characters. Stories are a familiar, powerful and persuasive resource to convey values, to teach emotion regulation, to promote learning content (e.g., spoken and written language, math, world knowledge), and transversal skills (e.g., SRL strategies) [23].

Even though some teachers are familiar with the term “self-regulation”, mostly regarding motor and attention control aspects, their SRL literacy is poor and there is a lack of the theoretical and operational knowledge on the concept [24]. In fact, specific training on the subject is scarce or non-existent in some countries, and more initiatives are necessary [25, 26]. The most effective initiatives are those with combined interventions, where children exercise skills and teachers train and apply guided practices based on the same principles simultaneously [1, 27].

As a result of these considerations, the second aim of our research was to study whether preschoolers who experienced an intervention with guided practices in SRL strategies (i.e., a story following SRL principles, SRL questioning, and authentic preschool tasks) used these strategies more often than those who experienced teachers’ autonomous practices.

### Intervention and assessment: The Dynamic Assessment of Self-regulated learning in Preschool method

Dynamic assessments are useful approaches to evaluate on-going processes like those which are applied by a learner while performing a task because, when successive learning states are examined over time, they provide a dynamic picture of the learning process [28]. Dynamic instruments are based on the assumption that the assessment is an open process, and respect the complexity of factors that influence the learning process, considering it as an unfinished product. Therefore, it is commonly seen as an assessment approach that is also an intervention, motivating the modifiability of the processes instead of their permanency [29]. Since the major objective of dynamic assessment is to identify the learner’s potentialities and difficulties, rather than obtaining a score, the objective is to uncover the individual’s learning potential [30, 31] and, therefore, it is considered an adequate approach to assess SRL skills. Moreover, a relevant role is given to adults that apply the assessment as they actively engage in the process, interacting with the child and highlighting the learning potential of the latter [32]. Considering preschoolers’ characteristics in terms of language acquisition and maturity of psychological processes, few instruments are available following the dynamic assessment principles. Those which are reliable and close to the aforementioned approach use digital tools [20, 33] and quantitative methods [34, 35], and others, although not assuming a demarked dynamic assessment approach, apply interactive procedures [36, 37]. However, none could provide robust results assessing the SRL cycle as a whole consistently, so they could not be adapted to other contexts.

Consequently, the Dynamic Assessment of Self-regulated learning in Preschool (DASP) method was designed to measure SRL skills in preschool children considering their developmental characteristics, a curricular infusion model, and the multidimensionality of the SRL process. The method structure follows a solid theoretical framework [18], and its reactivity effect is emphasized by the approach that is assessment and intervention at the same time [16]. It gathers different types of measures to fairly assess several data (e.g., self-report, observation, product of the task) while the child solves an authentic preschool task, in a given context and time. Previous studies using the DASP method have shown positive results assessing both overt and covert SRL processes in preschool children [38, 39], but its social validity was not investigated yet. Then, it is pertinent to understand teachers’ perceptions on the method’s utility and its feasibility in preschool daily practices regarding the design of research instruments that are ecological and socially sustained. In view of this, the third aim of the study was to assess the social validity of the DASP method with preschool teachers.

### The present study

The presented framework has contributed to the state of art on how preschoolers’ SRL skills can be fostered in school. Teachers’ daily practices have an important role concerning the opportunities given to children to make choices and experience learning challenges, but structured interventions are also an appreciated tool to develop SRL strategies. Some educational interventions with an SRL nature have presented good results, and when a solid theoretical concept supports the instruments (e.g., stories, activities), the intervention cohesion and validity is reinforced. To assess the SRL skills of preschoolers and, therefore, the efficacy of interventions to promote these skills, few measures are available, but the DASP method proved to be adjusted to context specificities.

We advocate that the novelty presented by this study fills a gap in the literature to design ecologic resources to foster SRL in preschool, considering it as a dynamic and multidimensional process. The fact that the educational intervention *Pipo and Mia*, *the magic knights* was designed including a story following SRL principles, adding an SRL questioning throughout the intervention sessions, and exercising children’s SRL skills with authentic preschool tasks, allowed to fully promote the strategies and processes applied in all the cyclical phases of the SRL model [18]. Furthermore, the intervention was implemented by teachers while engaging in the professional training *Practices to promote SRL in preschool*, which was also based on the same framework. In line with the background presented, and our mixed-method study objectives, two hypotheses were addressed:

*Hypothesis 1*: *After experiencing the aforementioned intervention*, *children’s use of SRL strategies (i*.*e*., *forethought*, *performance*, *and self-reflection skills) will increase*.
*Hypothesis 2*: *After the aforementioned intervention*, *children belonging to the Guided Practice group will demonstrate an increase in the use of SRL strategies (i*.*e*., *forethought*, *performance*, *and self-reflection skills) when compared to those in the Autonomous Practice group*.

Moreover, the fact that the DASP method was considered to evaluate the intervention efficacy, and it was applied by children’s teachers, reinforces the ecological concern of the investigation, even its usefulness and applicability to preschool practices needed to be studied. Thus, a third hypothesis was addressed:

*Hypothesis 3*: *Preschool teachers will find the DASP method to have acceptable social validity*.

## Materials and methods

### Participants

A convenience sample of 115 children that spoke Portuguese or Portuguese-Brazilian fluently participated in this study. Children were between 3 years and 5 months and 6 years and 3 months old (M = 4.9; SD = .70) and 53% were female. The participants attended state-owned preschools in three Portuguese counties: four schools in the center and two in the north of the country. Children belonged to 8 classes that had between 12 and 25 preschoolers. The countryside classes had less students than those in the city zone. The sample also included nine female preschool teachers with 27 years of professional experience, on average (SD = 9; Min. = 14; Máx. = 38). Children belonged to classes led by these teachers. Four teachers performed the *Guided Practice* group (N = 29 children), four teachers performed the *Autonomous Practice* group (N = 64 children), and one made a qualitative validation of the DASP method (cf. Procedures section).

### Instruments and resources

#### The Dynamic Assessment of Self-regulated learning in Preschool (DASP) method

**Fig 1:**
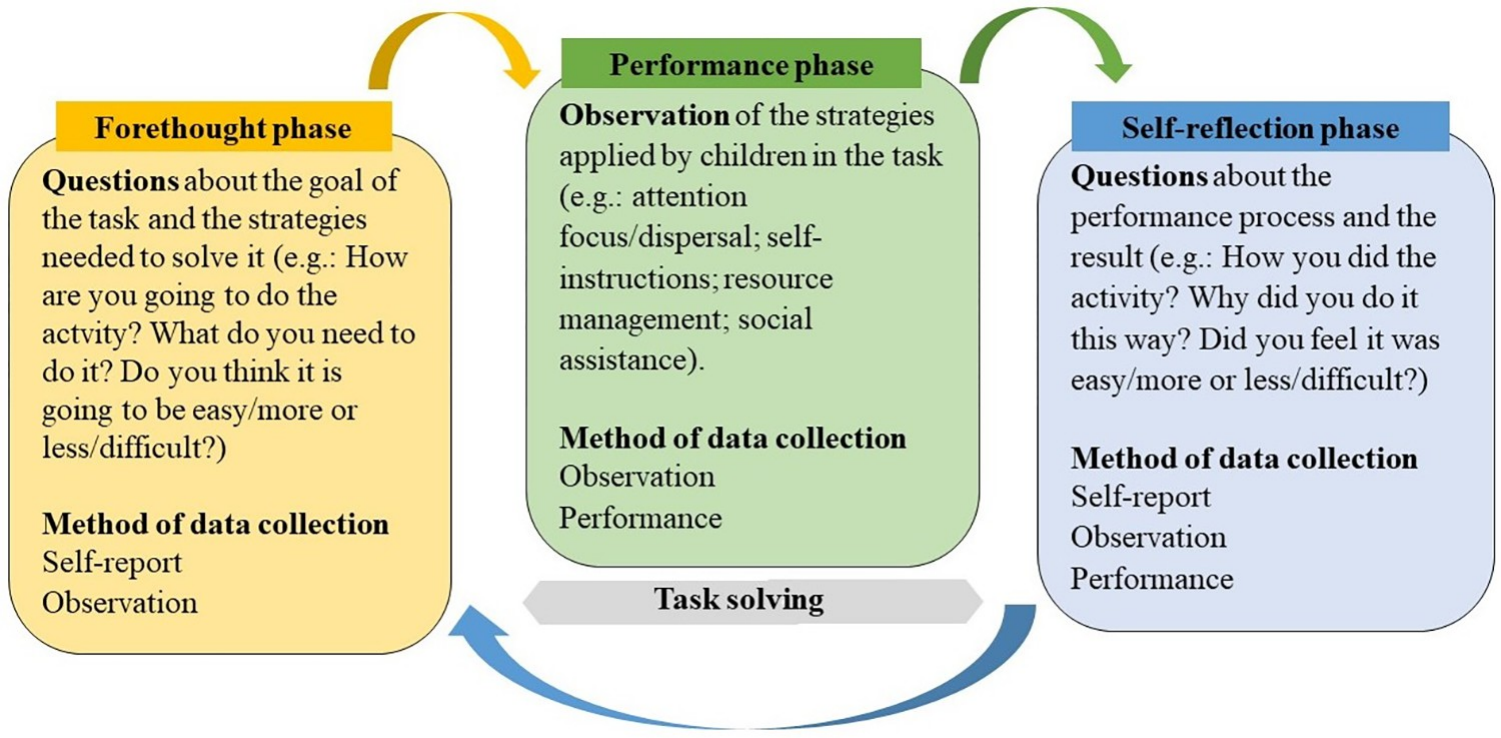
SRL model. Multimethod approach.

This method recreates a preschool learning situation, with a game-like format and a protocol of task interview adapted to preschoolers. The application allows for teachers to take notes on the children’s answers in a precise and systematic manner using the paper-pencil format of the instrument’s protocol, closely resembling a preschool daily routine. The method includes three parts: before, during and after solving a task, thus, matching the forethought, performance and self-reflection phases respectively [18]. Different types of measures to collect data are used in each phase, seeking to capture SRL processes from a dynamic, cyclical, and multidimensional perspective. The measures considered the developmental characteristics of the children [40, 41], such as their difficulty to speak about self-regulatory processes, so self-reported data is complemented by other sources, namely observation data and the product of the task, with the aim to triangulate the data. Accordingly, the multimethod data collection of the DASP method intends to assess the complexity of the SRL strategies and processes in a dynamic manner (Fig 1). Previous studies validated the method’s adequacy to assess SRL in preschool and described it in detail [38, 39].

**Fig 2:**
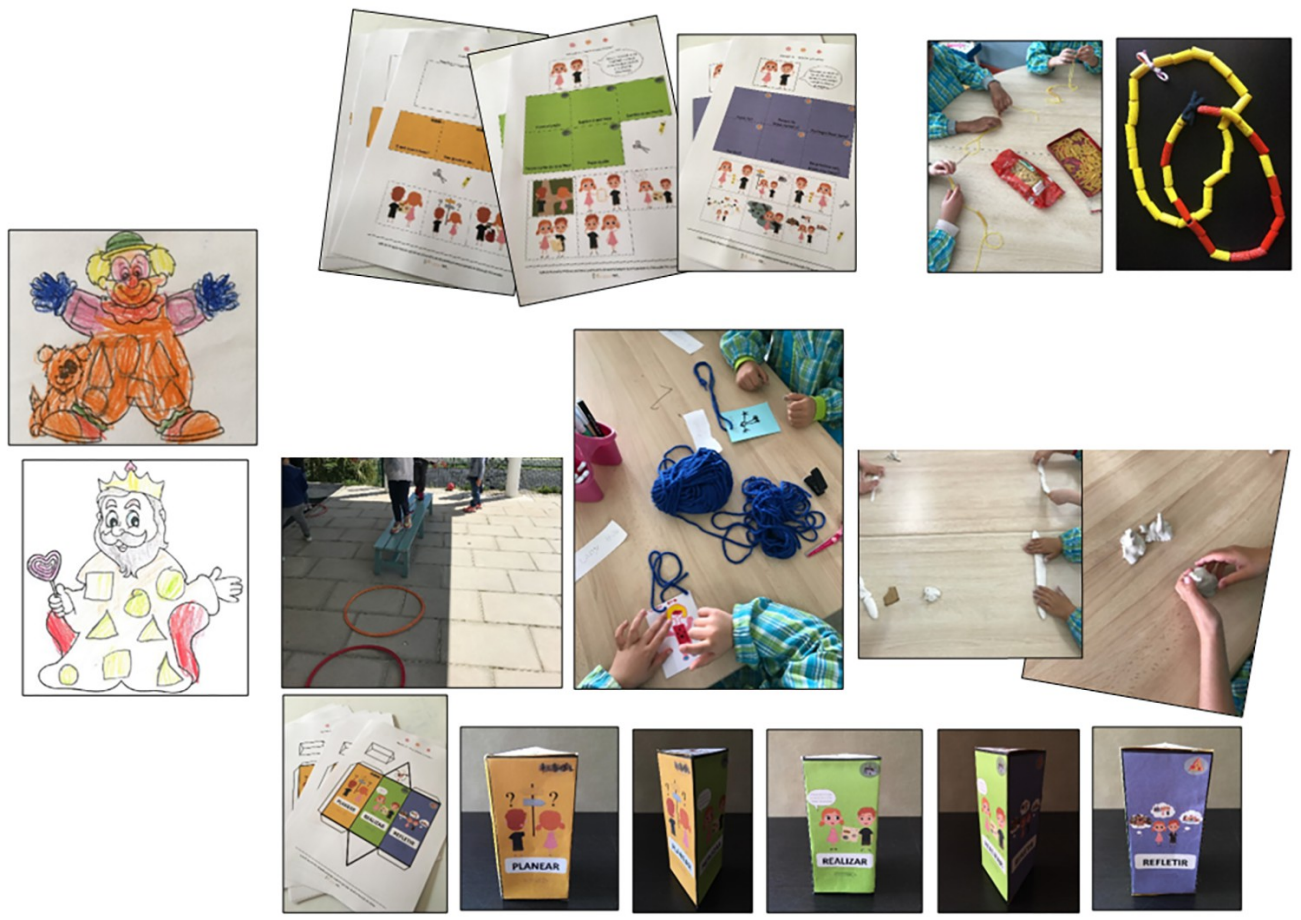
Authentic preschool tasks used in the research.

In this study, the tasks performed by children in the DASP method were authentic curriculum-based preschool tasks, considering specific developmental aspects, where children could feel the situation as resembling their daily life, mobilizing similar strategies and processes. Following the literature principles [42], tasks included four characteristics: (1) allowing to mobilize SRL strategies and processes applied in the specific context and while it is happening; (2) enabling children to plan, monitor and assess performance according to the tasks’ initial goals; (3) curricular content mastered by children was included, so previous knowledge could be fully exercised and the assessment results referred to SRL competency development and not to the unfamiliarity with the content of the task; (4) the type and the number of cognitive operations were according to the curriculum, the children’s developmental stage and the standard educational practices for this age, as the tasks were accessible to resolve, but sufficiently challenging to potentiate SRL competencies (e.g., solving problems). For instance, in the pre-test moment, we used a task that had been previously piloted and found to be appropriate for preschool children [38, 39] according to educational guidelines. It included content about geometrical figures (recognizing and replying), and numeric notions (count from one to three). It presented two clowns: a model on the left side of the page with three triangles, three circles, and two squares drawn as clothing patterns, and, on the right side of the page, there was a similar clown, but its clothes had no patterns. Children were instructed to follow the task instruction: "Draw the same number of triangles, circles, and squares in the clown on the right side." One of the task result is depicted on the left side of the Fig 2.

#### Teachers focus group interview

Regarding our preschool teachers’ sample size, a group discussion methodology moderated by the researchers was considered suitable to understand this target group’s choices, behaviors, and feelings. Their perceptions and the frequency with which they verbalize them allowed us better to integrate the research objectives with the participants’ voices [43]. Therefore, a semi-structured interview was especially designed for the purpose of this investigation. The main four questions aimed to identify teachers’ perceptions on the experience with the DASP method; to understand their motivational beliefs on the method; to identify beliefs about their performance and self-efficacy, and how they make the self-regulation process visible; and to recognize the beliefs of the method’s efficacy in children performance and behavior (e.g., The DASP method is useful to promote SRL? Why?; Which were the most relevant aspects in children’s answers regarding the forethought phase?). Our criteria for using this instrument were to collect qualitative assessments to help interpret this project’s outcomes and to inform future improvements [44, 45]. As secondary goals, we addressed: to reflect about the potentialities and constrains of the DASP method, and to identify changes in children and teachers along the process.

#### Intervention design

**Fig 3:**
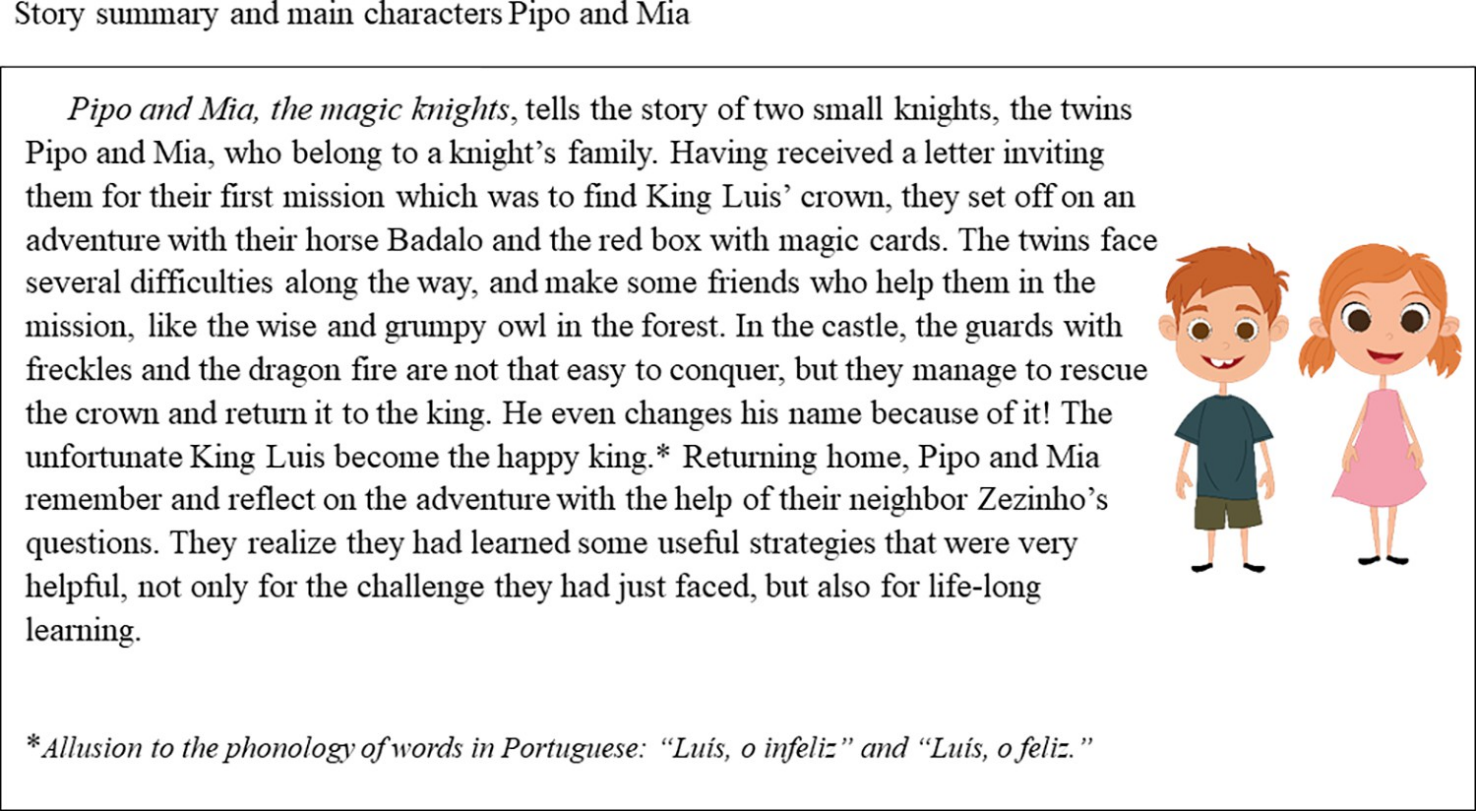
Story summary and main characters *Pipo and Mia*.

Educational intervention *Pipo and Mia*, *the magic knights*: the intervention was designed by a multidisciplinary team (i.e., Educational Psychology researchers, SRL experts, preschool teachers, and a designer), and delivered by teachers to their preschoolers. To reach the intervention goals, some methodologies suitable to foster SRL in preschool were used: a story following the SRL model in a ludic and playful way, SRL questioning, strategic explicit instruction, modeling, dual-coding, group dynamics, and positive feedback. The intervention active and engaging methodologies facilitated the learning process [46], stimulated children’s imagination, strengthened motivational factors, and increased preschoolers’ SRL skills, enabling the development of transversal learning skills (e.g., SRL and metacognitive strategies) while applying them to authentic preschool tasks. For instance, in activity 12, children were invited to make a triangular parallelepiped. Children had to cut, fold, and glue a printed sheet, and then assemble their magic prism. Each of the three parts had a figure and a keyword corresponding to each SRL phase. It should work as a personal self-regulatory resource, encouraging the generalization of the processes and strategies learned during the project to all preschool activities (the task result is at the bottom of Fig 2). For each thirteen intervention sessions, teachers followed a script (S1 File), reading aloud a story chapter to the class and challenging them to experience an activity, in a curricular infusion model. Children were led to explore personal, emotional, motivational, and metacognitive dimensions by helping the story characters (Fig 3).

**Fig 4:**
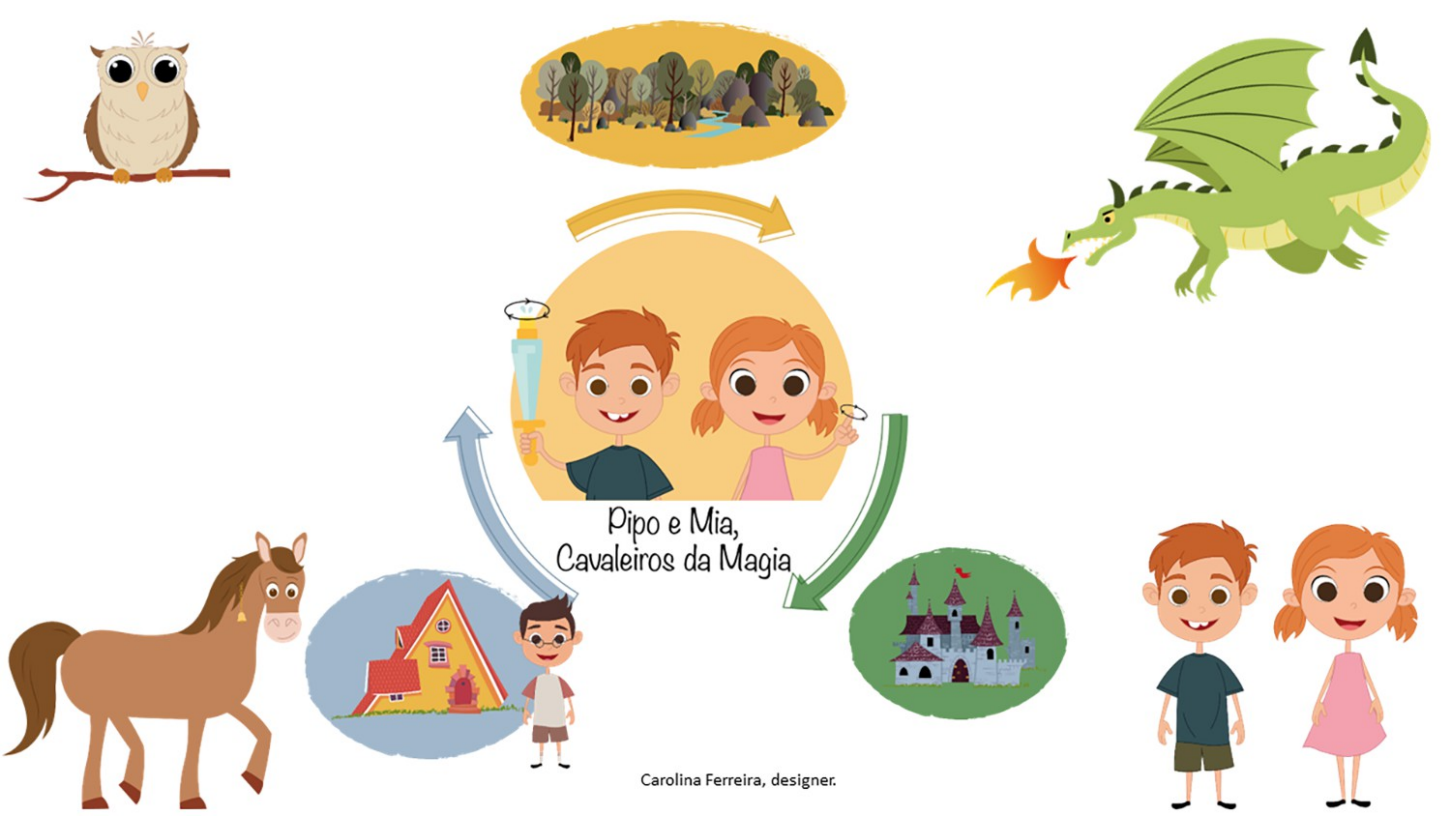
Illustrations of the educational intervention *Pipo and Mia*, *the magic knights*.

In preschool, since a strict program is not mandatory, the intervention flexible approach was welcomed, promoting both learning contents and transversal skills, and contributing to children’s integral development [8, 9]. Theirs developmental specificities were considered through the use of appealing and expressive images, since most children had not yet learned to read, and the characters’ names rhymed in the Portuguese language, facilitating phonology and memorization (Fig 4).

**Fig 5:**
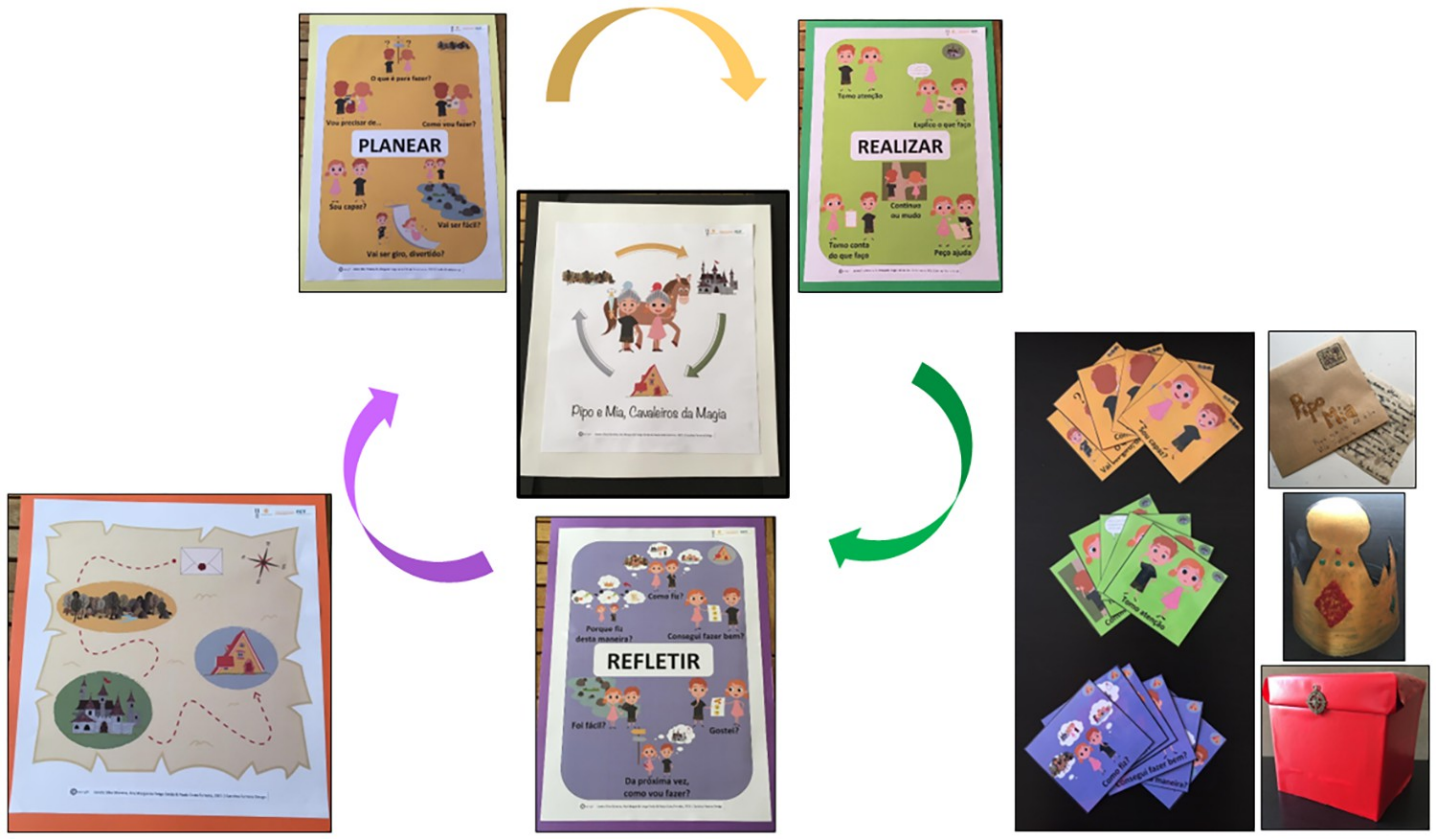
Materials used in the educational intervention *Pipo and Mia*, *the magic knights*.

The SRL questioning was specifically addressed through the magic cards in a red box carried by the characters (e.g., What am I going to do? What do I need? Pay attention. Ask for help. Was it easy? How am I going to do this next time?), and other elements helped children follow the characters’ paths in the adventure (Fig 5).

In the performance phase, one more session was included than the other phases allowing children more training, since previous study found that they tend to feel it more difficult [39]. The intervention overview is presented in Table 1.

**Table 1:** Intervention overview: Self-regulated learning phases, sessions, activities titles, and general goals.

SRL phase	Sessions and activities titles	General goals of the intervention
Introduction to the intervention/anticipation	1-My nickname	• To explore on the importance of self-regulating strategies in planning/performance/self-reflecting; • To promote the knowledge transferability of each SRL phase to other preschool activities; • To recall, integrate, and consolidate the SRL strategies learned.
Forethought	2-The sounds of the forest	
	3-Symbols for planning	
	4-Crossing the Cold River	
Performance	5-The king’s castle	
	6-The dragon	
	7-The poem	
	8-Symbols to perform	
Self-reflection	9-Magic knights	
	10-Math knights	
	11-Symbols to reflect	
Integration	12-An unforgettable adventure	
	13-The knight necklace	

Professional training *Practices to promote SRL in preschool*: preschool teachers participating in this study attend the professional training throughout the school year, completing 50 hours. The training was designed by SRL and Educational Psychology experts, concerning the needs of preschool context and literature advice [5, 12], aiming to lead teachers to reflect, learn and train practices to promote SRL in preschoolers, and to develop and improve resources to assess and intervene in this field (S2 File). It included thirteen online sessions with an SRL structure, aiming to promote the theoretical principles, and playing a solid approach with the other intervention resources, where direct work was developed between researchers and teachers, to discuss and practice some activities. Simultaneously, autonomous work time was developed by teachers corresponding to the educational intervention application with their classes in the preschool scenario.

### Procedures

Ethical issues were assured, and the research was approved by the Commission of Ethics and Deontology of the Faculty of Psychology of the University of Lisbon. Participants were recruited for this study between September 21, 2020, and November 3, 2020. Teachers’ informed consent to participate in the study was verbally obtained and witnessed by the researchers. Children’s parents were informed about the research and gave their verbal consent. Anonymous participation was guaranteed through an identification code. As for the research design, a pilot study with a mixed-method design was developed. Regarding the intervention’s efficacy assessment, and children development of the use of SRL strategies, quantitative methods were used to collect pre and post-test data of two intervention groups (i.e., the DASP method). Our aim was to compare two groups’ outcomes when experiencing the intervention with different educational methodologies. Specifically, the *Guided Practice* group listened to the story following SRL principles, trained the SRL questioning explicitly, and performed 13 authentic preschool activities proposed by the researchers. The *Autonomous Practice* group listened the same story but performed thirteen activities organized by their own teachers autonomously. The DASP method was applied before and after the intervention sessions (i.e., December/January and May/June) by teachers. The method was applied individually to each child during the daily preschool activities, inside the room while the ordinary activities were occurring at the same time with other children. Some material was available on the table (e.g., pencil, rubber, coloring pens, and the task sheet). The time of application lasted 10 minutes, on average. Afterwards, the DASP method protocols were evaluated by the researchers in terms of the participants’ answers, and the tasks were evaluated in terms of goal achievement.

As far as the social validity assessment is concerned, teachers’ perceptions on the DASP method were collected using qualitative methods (i.e., teachers’ focus group interviews) at the beginning and at the end of the intervention. The interviews were conducted online by the researchers following an appropriate script to attain the study’s goals. The interviews lasted 1h28m to 1h52m. One of the teachers worked as a preschool expert testing the DASP method with her class (i.e., 22 preschoolers), providing suggestions, and assessing the experience qualitatively, which supported the validity of the instrument. Since the intervention was not applied to this class, they were not included in the analysis.

The professional training was delivered by two SRL experts, and two Educational Psychology researchers selected due to their previous experience and knowledge related to SRL. To be properly prepared, the researchers practiced their competencies with roleplays supervised by experts. During the intervention’s delivery, the researchers followed a step-by-step session script, and attended weekly supervision meetings. Aiming to overcome difficulties and doubts concerning the pandemic, institutional, and educational issues, the team tried to find solutions that did not put the training program at risk, and could fit each context’s characteristics. The research procedures and timings are depicted in Table 2.

**Table 2:** Research procedures and timings.

		When		
		Before the intervention (pre-test)	Sessions 1–13	After the intervention (post-test)
What was assessed	Intervention efficacy	Children’ SRL skills assessment: the DASP method	Implementation of the intervention	Children’ SRL skills assessment: the DASP method
	Social validity	The DASP method assessment:	Professional training attendance	The DASP method assessment:
		Teachers’ focus group interview		Teachers’ focus group interview

### Data analysis

Children’s responses in the DASP method were categorized from 1 to 4 where a higher score (4) indicated a more complex strategy, the middle values indicated common strategies and the lower score (1) pertained to an irrelevant response or a non-response. An exploratory analysis for descriptive statistics with percentage results was run to explore the findings. Then, a statistical analysis was selected to compare means between the two intervention groups [47] using *IBM SPSS Statistics*, version 25.0 for Microsoft Windows 10. Scale diagnosis for missing values, outliers and normality distribution were computed. Missing values had little expression (< 5%). Regarding normality and outliers’ analyses, *Q-Q* plots evidenced a tendency for normal distribution for all outcome variables, suggesting that the outliers did not have a marked impact on the data distribution. Considering these results, mean differences were assessed through t-test for independent samples, and significant effects were considered for *p* < .05.

The tasks solved at pre- and post-test moments were assessed similarly to the previous data (i.e., 1—goal task not achieved; 2—goal task achieved).

Regarding the qualitative data, a content analysis was run to assess teachers’ perceptions collected in the interviews. The principles guiding the category construction were both deductive (e.g., utility of the DASP method to assess and promote SRL skills) and inductive (e.g., opportunity to reflect about practices). The category system and the operational definition was assessed by two experts. The procedure was conducted manually: a free reading of verbatim was done, and then, the main themes were detached and organized into categories and subcategories, looking for a coherent sense according to the study’s objectives and following Bardin’s guidelines [48].

## Results

Upon close examination of the results on the intervention’s efficacy and SRL strategies applied by children, this section presents the findings in light of the hypotheses, in three subsections: an overview of the results at pre (T1) and post-test (T2) for each SRL phases, and the evaluation of the task; a comparison of the intervention groups’ averages, and the outcomes resulting from the DASP method social validity.

### General improvements from pre- to post-test

**Fig 6:**
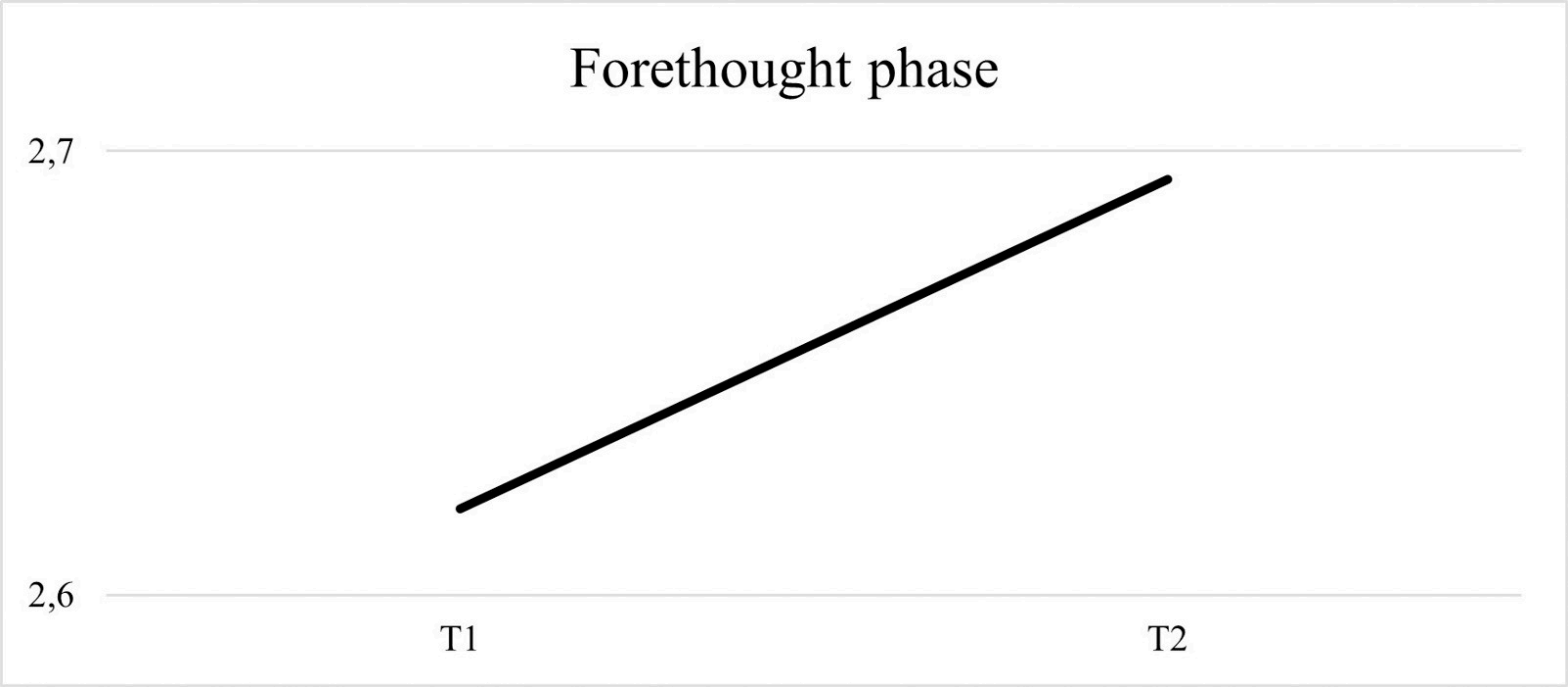
Results regarding the average of forethought strategies. Strategies used by children at the beginning and at the end of the intervention.

Regarding the average of forethought strategies used by children, Fig 6 shows an increased use from the pre-test to the post-test.

Detailed results concerning this phase categories are summarized in Table 3.

**Table 3:** Results concerning the use of specific forethought strategies at the beginning and at the end of the intervention.

Categories of the forethought phase	Indicators	T1	T2
Goal identification	The child does not identify the goal or asks to confirm it.	27%	14%
	The child identifies the goal.	72%	86%
Organizing and transforming	The child does not anticipate or anticipates only resources.	69%	62%
	The child anticipates strategies.	31%	35%
Establishment of performance goals	The child describes the action or points the goal in a non-verbal way.	87%	94%
	The child does not describe or anticipate the action.	13%	6%
Self-efficacy perception	The child says that they can do the activity.	82%	90%
Interest of the task perception	The child says that the activity is going to be interesting, funny.	91%	95%
Perception of the task´s difficulty	The child says that the activity is going to be easy.	80%	81%

The analysis shows a general improvement in the use of forethought strategies. Specifically, in the category *Goal identification*, where there was a 14% improvement in the number of children that could identify the goal of the task at the beginning when compared to the end of the intervention. Furthermore, although smaller, an increase can also be observed about the use of organizing and transforming strategies. That is, more children could begin to anticipate strategies (e.g., “I need to paint”; “I need to pay attention.”), and not only resources to solve the task (e.g., “I need a pencil”; “My pen.”). As for the category *Establishment of performance goals*, corresponding to the question “How are you going to do the activity?”, this seems to be one of the most difficult within the scope of planning skills, as children struggled to differentiate it from the category *Organizing and transforming*, confusing the anticipation of resources and strategies and the anticipation of the action. Yet, an increase of 7% can be observed in the number of children who could describe their intentions (e.g., “First, I am going to… Then, I will…”), even if they pointed their goal in a non-verbal way. Concerning the *Self-efficacy perception*, children felt more able to solve the task at T2 with an increase of 8%, as well as in the *Interest of the task perception*, where there was a slight increase on the number of children that felt the task more amusing at post-test. Finally, the category *Perception of the task’s difficulty* also presented a small increase on the number of children that felt the task easier at the end of the intervention.

**Fig 7:**
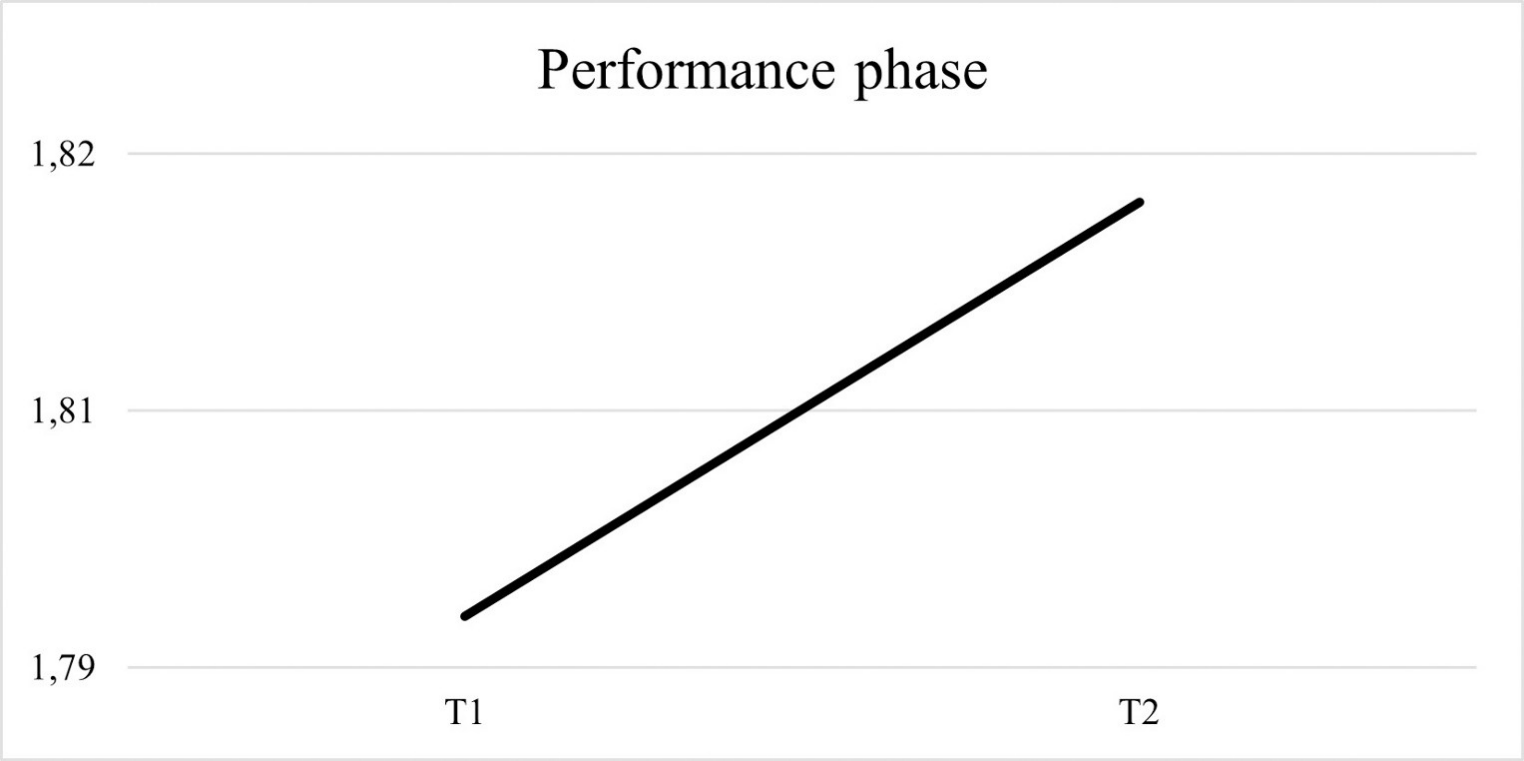
Results regarding the average of performance strategies. Strategies used by children at the beginning and at the end of the intervention.

Regarding the average of performance strategies used by children, Fig 7 shows an increased use from the pre-test to the post-test.

Detailed results concerning this phase categories are summarized in Table 4.

**Table 4:** Results concerning the use of specific performance strategies at the beginning and at the end of the intervention.

Categories of the performance phase	Indicators	T1	T2
Attention focusing	The child interrupts the procedure or avoids the task.	22%	6%
	The child maintains his/her attention on the task.	78%	94%
Self-instruction	The child uses audible self-speech.	41%	37%
Resource management and monitoring	The child manages some resources towards the goal.	86%	89%
Seeking social assistance	The child asks for help.	13%	5%

Higher results are shown in two categories: *Attention focusing* and *Resource management and monitoring*, and lower results are presented for the other two categories: *Self-instruction* and *Seeking social assistance*. The totality of the results represents positive indicators of children internalizing performance strategies from the beginning to the end of the intervention. Specifically, about attention focusing, the results show an increase in the number of participants that could maintain their attention while solving the task. Similarly, at post-test, more children used strategies to manage and monitor their performance, which was observed through children’s expressions, such as “Is right here” or “Now, I need…” or by changing the color of the pen or using the rubber to erase and redraw. Moreover, the findings on the use of self-instructions (i.e., the child uses audible self-speech/the task is solved in silence) show a descending value, meaning that less children thought aloud during the task at T2. On the other hand, 8% less of children asked for social assistance at post-test (i.e., the child asks for help), which could suggest that children performed the task more autonomously at the end of the intervention, asking for teacher’s help less frequently.

**Fig 8:**
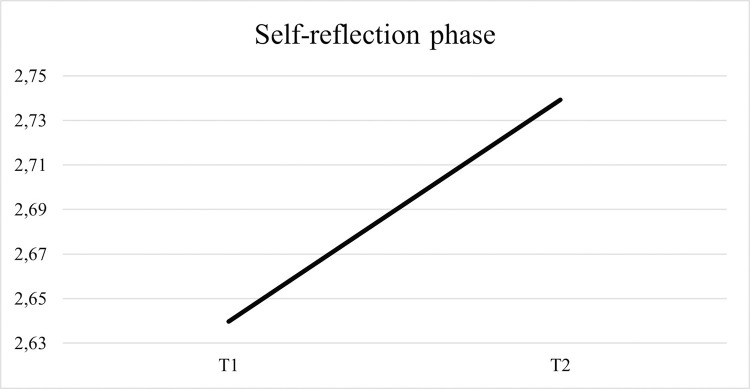
Results regarding the average of self-reflection strategies. Strategies used by children at the beginning and at the end of the intervention.

Concerning the average of self-reflection strategies used by children, the Fig 8 shows an increase from the pre- to the post-test.

Detailed results concerning this phase categories are summarized in Table 5.

**Table 5:** Results concerning the use of specific self-reflection strategies at the beginning and at the end of the intervention.

Categories of the self-reflection phase	Indicators	T1	T2
Descriptive assessment	The child mentions general aspects of the task performance.	55%	48%
	The child mentions specific aspects of the task performance.	43%	51%
Strategic approach assessment	The child does not name strategies.	14%	10%
	The child names strategies to perform the task, achieving the initial goal or other goal.	86%	90%
Efficacy assessment	The child says that they did the activity well.	80%	88%
Causal attribution	The child does not names causes for the result nor names external causes.	38%	16%
	The child names internal causes for the result.	62%	84%
Perception of the task’s difficulty	The child says that the activity was easy to do.	83%	87%
Affective reaction	The child says that the activity was enjoyable.	95%	98%
Self-satisfaction	The child says they are happy with what they did.	89%	96%
Adaptive/defense inferences	The child does not respond or make changes.	60%	58%
	The child only identifies mistakes.	5%	3%
	The child names cognitive or metacognitive strategies to improve the result.	35%	39%

Relevant results emerge from the observations made through the DASP method, like the number of participants who could explain specific aspects of the task performance that increased at posttest (i.e., *Descriptive assessment* category corresponding to the question “Can you explain to me how you did it?”), meaning that some children turned their vague explanations (e.g., “I did like I should.”) into more complete and concrete interpretations (e.g., “First I looked to the forms, then I chose a color, after I drew the triangles.”). Regarding the *Strategic approach assessment*, corresponding to the question “Why did you do it this way?”, a change was observed in the number of children who started the intervention by not naming strategies to justify the result of their task in favor of an increase, although small, of participants who named strategies to perform the task, achieving the initial goal or other goal, at the end of the intervention. An increased percentage is also shown about the efficacy reported by children after the task was solved, where more participants assessed it positively (i.e., The child said that they did the activity well). This subject can be related to a reinforcement of the children’ self-confidence at post-test due to more familiarity with the type of tasks presented in the measure times [11, 49]. Concerning de category *Causal attribution*, there was an expressive increase of 22% on the number of children who named internal causes for the task result, at T2. This was the major difference found between the beginning and the end of the intervention, as the number of children who did not name causes regarding the result or name external causes decreased in favor of a greater awareness of participants about the reasons for their performance. The task´s difficulty was also perceived as more reachable at the end of the intervention, where 4% more of children felt the task was easy to perform. Additionally, more participants reported a positive perception about the task, answering “Yes” to the question “Did you enjoy the activity?” Children’s perception of self-satisfaction about their performance also increased 7% from the pre to the post-test. In addition, in the category *Adaptive or defense inferences*, uncovered by the question “How are you going to do this activity next time?”, more children could name cognitive or metacognitive strategies and anticipate improvements to the task at post-test. Although the increase is small (4%), it is very relevant in theoretical terms concerning the growing metacognitive awareness at preschool age [10, 50, 51].

Furthermore, regarding the achievement of the tasks’ goals, there was an expressive increasing on the number of children who achieved the goal at T2: 46% to 72%. This result suggests that children benefited from the intervention and, at the end of it, most of the participants were able to solve the task, thus attaining the goal requested by the instruction.

### Comparing the intervention groups

Seeking to answer the *Hypothesis 2*, the findings are presented below with the two group’s performance cross-analysis, at the beginning and at the end of the intervention, for each SRL phase (Table 6). Concerning the forethought phase, results show that, on average, participants of the *Guided Practice* group used more strategies at the beginning and at the end of the intervention than those of the *Autonomous Practice* group. At both time measures the difference was significant. In this phase, there was a big effect size between groups at T1 and T2. Specifically, our results indicate that the groups significantly increased their use of forethought strategies, benefiting from the interventions to promote planning skills, both when belonging to a fully guided SRL intervention and when teachers promoted SRL strategies in a more autonomous manner.

**Table 6:** Descriptive statistics of SRL phases concerning the two intervention groups, at the beginning and at the end of the intervention (values rounded to the second decimal place).

		Guided Practice	Autonomous Practice	T-test for independent samples					Cohen’s *d*
		M (SD)	M (SD)	t	df	Sig. (2-tailed)	Mean difference	95% CI	
Forethought	T1	2.82 (.32)	2.53 (.33)	3.91*	91	.00	.29	[.14, .43]	.89
	T2	2.89 (.24)	2.61 (.33)	4.16*	91	.00	.29	[.15, .42]	.97
Performance	T1	1.85 (.2)	1.81 (.19)	.71*	91	.48	.03	[-.55, .12]	.20
	T2	1.89 (.12)	1.83 (.28)	1.31*	91	.2	.06	[.04, -.03]	.27
Self-reflection	T1	2.67 (.21)	2.65 (.3)	.41**	77.18	.69	.22	[-.09, .13]	.07
	T2	2.85 (.15)	2.71 (.3)	2.96**	89.99	.00	.14	[.05, .23]	.59

*Equal variances assumed.

**Equal variances not assumed.

About the performance phase, results show that, on average, participants of the *Guided Practice* group used more strategies at the beginning and at the end of the intervention than those of the *Autonomous Practice* group. However, in both times the difference was not significant, meaning that the intervention training may not be the only reason for the improvements.

Regarding the self-reflection phase, and similarly with the other SRL phases, on average, participants of the *Guided Practice* group used more strategies at the beginning and at the end of the intervention than those of the *Autonomous Practice* group. At the first-time measure, the difference was not significant, but it was significant at the second time measure (i.e., medium effect size). These results suggest that the participants benefited from the intervention to promote SRL strategies, particularly those belonging to the group where SRL guided practices where exercised.

### Evaluation of the task

The results about the achievement of the goal task at T1 and T2 for the two intervention groups can be analyzed on Table 7. Results show that, on average, more participants of the *Guided Practice* group achieved the goal of the task at the beginning and at the end of the intervention than those in the *Autonomous Practice* group. At pre- and post-test, the difference was significant, and the effect size was medium, suggesting that children benefited from the intervention, especially those belonging to the *Guided Practice* group.

**Table 7:** Descriptive statistics concerning the achievement of the goal task concerning the two intervention groups at the beginning and at the end of the intervention (values rounded to the second decimal place).

	Guided Practice	Autonomous Practice	T-test for independent samples					Cohen’s *d*
	M (SD)	M (SD)	t	df	Sig. (2-tailed)	Mean difference	95% CI	
T1	1.62 (.45)	1.39 (.49)	2.09*	91	.04	.23	[.01, .45]	.49
T2	1.86 (.35)	1.66 (.48)	2.33**	72.29	.02	.21	[.03, .38]	.48

*Equal variances assumed.

**Equal variances not assumed.

### Assessing social validity of the DASP method

**Fig 9:**
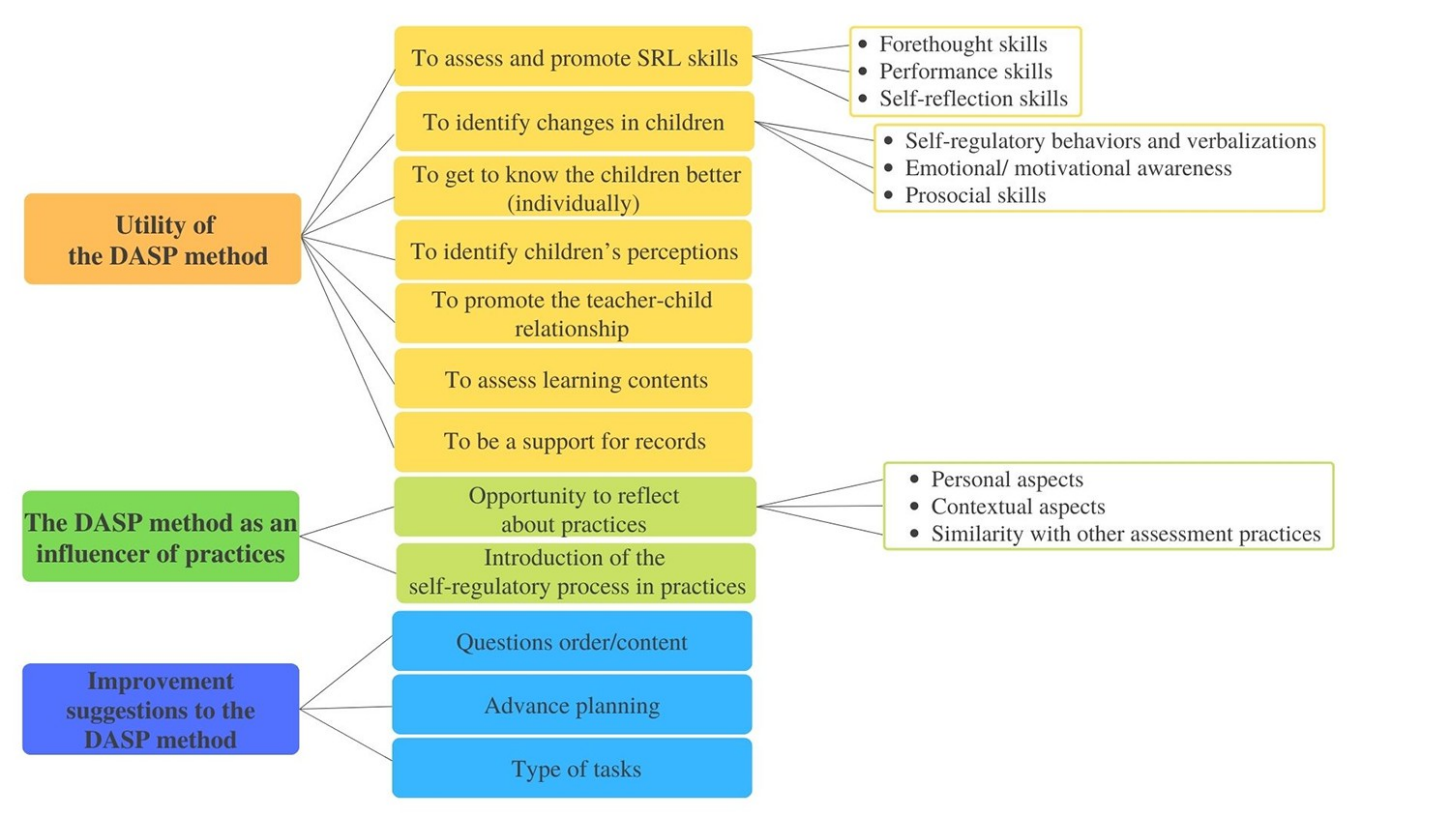
Scheme of categories. Content analysis of the teachers’ interviews about the DASP method application.

Aiming to answer *Hypothesis 3*, a scheme of categories was highlighted from the analysis of teachers’ verbatim (Fig 9).

**Fig 10:**
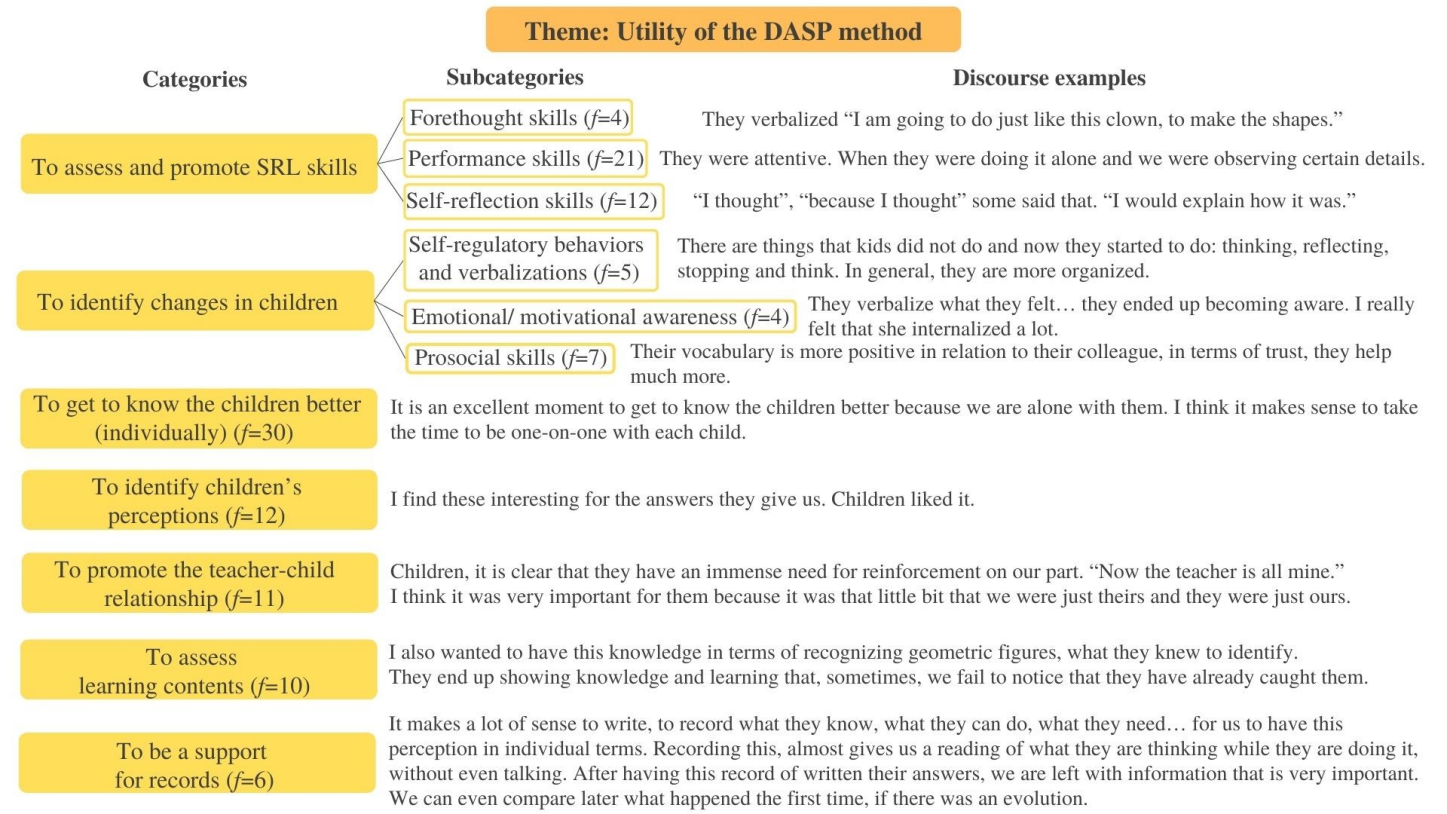
Theme utility of the DASP method. Categories, subcategories, frequencies, and teachers discourse examples.

Three main themes emerged from the analysis: *Utility of the DASP method*, *The DASP method as an influencer of practices* and *Improvement suggestions for the DASP method*. The first included several possible uses given to the method in teachers’ daily preschool practices. The categories were organized according to self-regulatory skills that can be assessed with the instrument; numerous subjects related to children (i.e., identify changes, to get to know them better, and to identify their perceptions); the teacher-child relationship; other aspects about the learning process (e.g., to assess learning contents), and the potentialities included in the method to make convenient annotations to children’s global assessment. Clearly, teachers identified a set of useful areas to apply the method, to both assessment and intervention issues. Inclusively, some indicators identified in teachers’ speech go in line with the instrument’s theoretical framework related to the SRL phases (i.e., to assess and promote SRL skills: forethought, performance and self-reflection skills), like when teacher 5 (t5) said: “in that phase when they are performing by themselves and we are watching, made us look to certain details” and “it was important for them to reflect, so they could be aware of what they had done” (t1 and t2). Moreover, teachers also identified changes in children between the measurement times, showing more evidence of self-regulatory behavior and verbalizations (“Some things that they did not verbalize, and now they internalize it.” t6 and t8), emotional/motivational awareness (“I think, now, children are more aware and motivated about what they are doing.” t8), and prosocial skills (“You can do it, you are able to do it!”; “They became better friends by supporting each other.” t3 and t7). Fig 10 includes the frequencies of each category (*f* is the number of times the subcategory was verbalized) and some teachers’ discourse examples.

**Fig 11:**
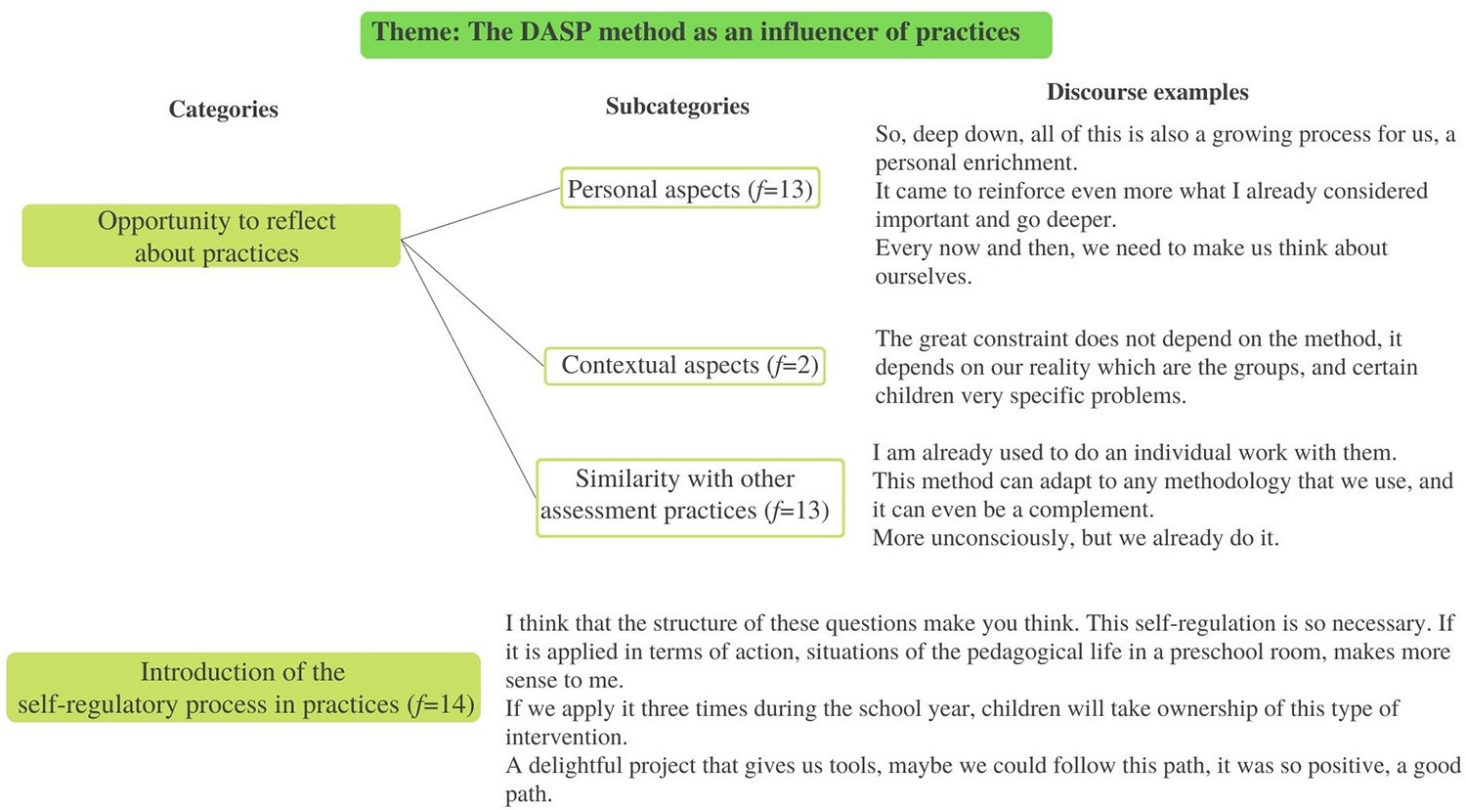
Theme the DASP method as an influencer of practices. Categories, subcategories, frequencies, and teachers discourse examples.

The second theme *The DASP method as an influencer of practices*, referred on how the use of the method can impact preschool teachers’ usual practices. The opportunity given through the application of the method to reflect on teachers’ lives, both personally and professionally, and their choices was included, as well as reflecting about contextual constraints and parallel assessment practices. During the interviews, references were made to the personal aspects that were improved from the reflection exercises inspired by the method (e.g., “I am looking back… there is a growth; it is a professional enrichment because it made me reflect.” t5), and some school limitations were identified that may difficult the repeated use of the method (e.g., “our reality makes it difficult, big groups.” t8 and t9). Interestingly, several similarities were found between the DASP method and other assessment practices already applied, which may facilitate the continuity of the instrument application after the professional training. Furthermore, the application of the method revealed the pertinence of including the self-regulatory process in daily work and teachers seamed available and interested to introduce the SRL principles in their ordinary practices (e.g., “If we apply this method in the beginning of the year, in the middle, and in the end, we may notice an evolution.” t1 and t6). Fig 11 includes the frequencies and categories of the theme, and some teachers’ verbalizations.

**Fig 12:**
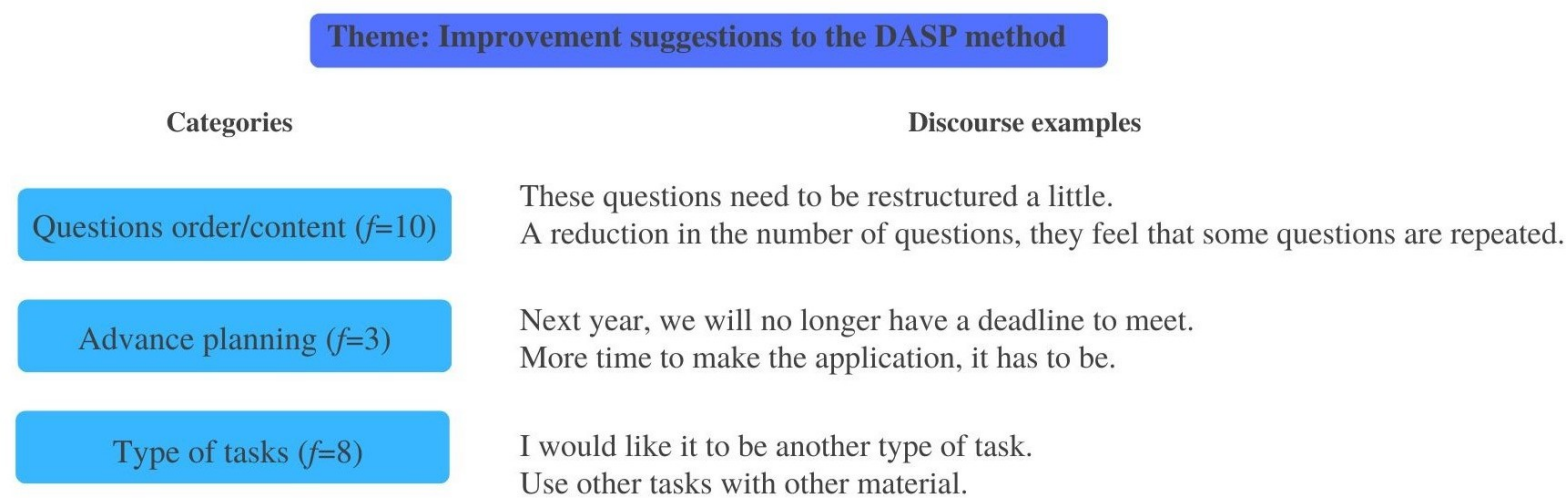
Theme improvement suggestions to the DASP method. Categories, subcategories, frequencies, and teachers discourse examples.

The third theme was related to improvement suggestions made by teachers to the DASP method, where potential enhancements were suggested, aiming to make the instrument more suitable to preschool practices. Indicators are related to the SRL questions in the instrument protocol, the planning of the application (i.e., plan well in advance to organize it better with other activities), and the tasks used in the instrument application during this research. Teachers made a critical analysis of how the method was used in the research context, identifying some issues that should be improved to a truly ecological application, considering preschoolers’ developmental characteristics (i.e., “We need to have more freedom, and not be so focused on the protocol.” t4). Their enrollment in the discussion reinforced the pertinence of the instrument and the willingness of teachers to continue to use it (“It takes time, but when we want, we can do it by giving it the priority.” t8). The upgrade that was referred more frequently was the questions’ order and content, considering that some sentences were similar, and children may have struggled to differentiate them. In teachers’ opinion, those aspects may have contributed to children’s decreasing interest in the experience, especially in the self-reflection phase (“In daily practices we cannot do some things like that, it seems too repetitive.” t1, t2 and t7). Fig 12 shows the categories frequencies of the aforementioned theme, and some teachers discourse examples.

## Discussion

### Preliminary findings on the educational intervention *Pipo and Mia*, *the magic knights*

Regarding the increase on the use of SRL strategies in the three SRL phases after the intervention, mainly in terms of the complexity of the strategies used by children, the *Hypothesis 1* was answered affirmatively. Concerning the highest results presented by the *Guided Practice* group, the *Hypothesis 2* was partially sustained. Specifically, about the forethought phase, the major differences were related to the goal identification, meaning that more participants could identify it at post-test. Improvements regarding the forethought strategies were also found in other studies [21], but our approach allowed to relate this process data with the product of the tasks solved by children, that presented a growth in the ability to achieve the aim asked by the instruction, suggesting that children benefited from the intervention at both levels. This finding is relevant concerning children metacognitive development and, to what we know, preschool investigations usually do not include this type of approach [52]. Other signs of a more accurate metacognitive awareness in the forethought phase were found in the ability for children to anticipate what they needed to solve the task, and how they meant to perform the action. Teachers reported children’s difficulties to discriminate some questions, but this fact reinforce the importance of providing more opportunities to train and rehearse different teacher-child interactions to understand how certain conditions may promote children’s zone of proximal development [53, 54]. Concerning the emotional perceptions of the forethought phase, the results were higher at post-test, which is in accordance with the literature suggestions, naming that previous successful experiences and practice (i.e., pre-test moment and the intervention sessions) tend to reinforce such perceptions [11].

The findings on the performance phase suggested the children became more autonomous and showed a greater understanding on the management of resources and monitoring after the intervention. Other study had found positive results regarding this phase [3] but, as far as we know, any other intervention assessed the performance strategies in a detailed manner as we did. Although the improvements observed, the results were not significant probably due to the difficulty felt by children, as previously pointed by other studies [39]. Since this is the least externally guided phase, more and specific training opportunities seems to be needed so that a progressive internalization of the self-regulatory processes occur and children learn to monitor their own learning [28].

Concerning the self-reflection phase, an increase can be observed in all categories where children presented evident signs of a self-regulatory internalization and strategical behaviors, which suggest a metacognitive awareness when reflecting about the task [10]: children mentioned more specific aspects when describing their performance, they achieved the task goal naming the strategies used, and they assumed more personal causes for the result achieved (instead of external). As expected, the previous successful experiences reinforced the efficacy appraisal and the emotional responses (i.e., the perception of the task’s difficulty, the affective reaction, and the self-satisfaction) that increased positively [11, 49]. Finally, although other studies found the adaptive/defense inferences question difficult in the self-reflection phase [39], in this investigation an increase was observed on the number of participants who could name cognitive or metacognitive strategies to improve the task next time, reinforcing the argument that training opportunities and reflection exercises are valuable resources to develop children metacognitive awareness [50, 51].

### Social validity and potentialities of the DASP method

Considering the qualitative results, the study’s *Hypothesis 3* was answered affirmatively. The three themes that emerged from teachers’ discourse relevantly contributed to the advances of this investigation. Firstly, teachers identified several utilities for the DASP method recognizing that it concurs to know children better and help them to personalize their teaching practices; they could pay close attention to what children know, what they do, and what they are capable of, that sometimes go unnoticed in group moments. Therefore, the DASP method offers new possibilities to complement already existing observational instruments to identify children’s competencies (e.g., CHILD; [55], adding the chance of intervening on the learning situation due to its dynamic approach. The individual knowledge about each child turned to be relevant, not only to detect their needs and strengths, but also to identify their changes over time [30]. About the assessment of children’s knowledge, teachers reported that, with this method, they could evaluate transversal competencies (e.g., self-regulated learning), and learning contents too (e.g., numerical notions, geometrical figures, fine motor skills), representing a major difference regarding other instruments that only assess SRL skills [36, 55]. Though it was not an objective of the instrument, teachers could assess children learning transfer, identifying what they have learned and how they mobilize it to other areas (“They apply the strategy in other activities.” t7). This fact could probably be observed in other studies with a similar approach [37], but our study more complex design provided teachers’ a reflection exercise, allowing them to identify the transferability of the intervention training, which is not easy to achieve [56]. Another useful topic of the DASP method goes in line with the literature [53, 57]: the teacher-child interaction has an important impact on the development of young children’ self-regulation skills and this area should be specifically addressed in teacher training programs. In fact, our participants felt that teacher-child emotional and social relationship got strengthened and may be reinforced with the continuous application of the instrument. Moreover, teachers referred to children’s perceptions on the assessment moment as they felt the approach amusing, they were interested and enrolled in the learning experience, which proved that the motivational dimension of the learning process was reinforced and the importance of using ludic and appealing materials to assess and promote preschoolers’ SRL competencies [7, 19, 33].

The teachers’ speech revealed a set of categories related to the self-reflection opportunities conveyed by the instrument. In a variety of ways, they said: “I am stopping and thinking about what I do, what I choose to do with children, and who am I as an education professional in this stage of my career.” The findings suggested that having participated in this investigation, teachers could realize how they can improve their own practice, rethinking, rearranging, and updating their routines with new ideas and specialized training. Therefore, we defend that the reflexive component of the DASP method was potentiated in this study by the group interviews, turning the application into a formative approach for both children and teachers. Numerous of the previous described aspects were also referred by the teacher who did not apply the intervention, reinforcing its validity. Hence, the method’s potentialities were supported, and it proved to be worthy of further investigation on its own, so it can be used even when it is not integrated into an intervention.

Lastly, a reflection should be done on the DASP method’s validity content. Even though the instrument structure was theoretically grounded [16, 18], teachers perceptions allows us to understand that the tasks instructions can be optimized, as well as the items construction with regard to children’s difficulties [7]. Consequently, some adaptations can be considered related to the questions’ order, concurring to a truly practical and ecologic utility of the instrument in daily preschool practices. For example, in forethought phase, children struggled to differentiate the anticipation of resources and strategies (i.e., *Organizing and transforming* category) and the anticipation of the action (i.e., *Establishment of performance goals* category). To maintain the conceptual structure, categories should not be suppressed, but they can be reorganized to make the formulation clearer. Another teachers’ suggestion to improve the DASP method is the advance planning of the application. In this case, the research had established timings, but once the method becomes part of the continuous, autonomous, and independent teachers’ practices, they can settle the adequate time and conditions when planning annual, quarterly or monthly. Seeking to answer to other studies suggestions, we advocate that different spaces can be used for the DASP method application (e.g., offices, quiet rooms outside the preschool room), since it is uncomplicated to apply and no special equipment is needed [7]. Thus, the individual time with each child can be considered more frequently and with quality conditions.

### Limitations and future directions

Although this was an exploratory study, it used a small convenience sample, without randomization. Moreover, different sized intervention groups and classes were used, which meant management differences, and a pure control group is missing. The sessions’ application was not fully assessed because only teachers’ self-report data was collected, and protocol compliance checklists were not included. Therefore, future studies should contain those absent features, and consider cross-validation measures regarding the sessions’ development and the changes occurred in teaching practices [58]. Other procedures should also be pondered to assess the intervention efficacy, such as observation of teachers’ practices and perceptions [26]. A follow up measure should also be considered. These findings are an interesting avenue to pursue, and other researchers could continue to explore the intervention’s potentialities with different approaches and activities. However, the positive results should be interpreted with caution because not all of them were due to the intervention, as several changes happen due to children’s natural growth and maturation in stimulating learning environments [21]. We also are aware that teachers’ work derives from different pedagogical models, and other factors such as different levels of training on SRL may influence their practices [56, 58]. Since changes of beliefs and practices are transferable but time-consuming processes, longer interventions may be considered to determine strategies to potentiate reflexive practices, achieving more consistent results.

### Implications for practice, research, theory, and policy

Considering practical implications about the intervention and considering that SRL skills are transversal to content areas, it can be remade or reused with different activities following teachers’ pedagogical intentionality and practices improvements, giving children opportunities to exercise their self-regulation in degrees of freedom adapted to their own capacities [38, 59]. Beyond the research context, it allows a closer adaptation to each class specificities, meeting children’s interests and needs. The intervention can be considered as part of a set of tools to promote SRL in preschool, exploring other SRL resources in an autonomous manner (e.g., games, digital tools). The intervention’ story can be split in different chapters, so that the story supports other SRL opportunities to promote social interactions, co-regulation and shared regulation socially [52, 60].

Regarding the practical implications of the DASP method, it can be applied with different tasks, for several educational purposes (e.g., baseline, monitoring, intervention programs, etc.), and professionals (e.g., teachers, psychologists, early intervention technicians). The fact that authentic preschool tasks are used, makes the approach meaningful to children and provides ecological validity to the method [17, 61], which is underlined by the fact that teachers mentioned their intention to reuse the method according to learning goals beyond the investigation. The interaction fostered by the instrument gives professionals an opportunity to get reciprocal feedback in the interaction, helping children develop SRL strategies, and professionals to customize the approach to individual learning potentials [30, 31]. Moreover, for research purposes, the DASP method seems to be an appropriate measure to collect repeated measures allowing to track progress in SRL [7] and enabling longitudinal intervention designs [62].

Concerning the practical implications of the professional training, few similar opportunities are available to support the development of teachers’ literacy and competencies about SRL [25, 26] and it is a venue to pursuit in terms of research. The structure of the training used in this research project is advantageous because it is digital with remote supervision, also adequate for a face-to-face format, and allowing a person-centered approach [56]. In terms of duration, the format is flexible enough to be shorter than the version that was used in this study and could be followed up on, in future research designs.

At the theoretical level, this study proved to be beneficial to consider the three self-regulatory phases, which was different from other studies that considered only one part of the self-regulatory cycle (e.g., [36]). Our option contributed to understand how SRL complexity works, regarding the continuity between the self-regulatory phases, processes, and dimensions.

At the policy level, it should be highlighted that preschool is a cycle of education where learning goals can be promoted according to children’s characteristics and personal interests because there is time and flexibility [2, 8, 9]. In this study, plastic and ecological educational resources were made available which can support SRL approaches where the individual development can be respected [63]. In parallel, since the SRL development is influenced by several factors and some children the same age performs competencies differently, it is important to cultivate practices where their autonomy and efficacy are worked through processes (e.g., problem solving) instead of seeking only for the result [11]. It would be interesting to continue to investigate how Educational Psychology can support the development and monitoring of individual learning paths [64]. Policy guidelines refer planning and assessment processes as a pedagogical support to help children in regulating their learning experiences [65], reinforcing the pertinence of this research. To close, these implications should be analyzed considering each country political and social systems because they influence the functioning of educational institutions and its daily routine.

## Supporting information

S1 File 4^th^ activity intervention script. Activity approaching the forethought phase.
(PDF)

S2 File Professional training contents. (PDF)
