# Supplementary material for: Fostering self-regulated learning in preschool through dynamic assessment methodologies
Source: PLoS One. 2024 Mar 21;19(3):e0298759. doi: 10.1371/journal.pone.0298759 (PMC10956879; doi:10.1371/journal.pone.0298759)
Supplement: S1 File — Activity approaching the forethought phase. (PDF) [file pone.0298759.s001.pdf]

Supporting information 1. 4th activity intervention script (forethought phase)

	**Activity 4 – "Crossing the Cold River"**
**Preschool content learning areas included in the activity**	Personal and social area;<br>Expression and communication area: motor expression domain;<br>World knowledge area.
**Type of activity**	Large group.<br>Activity of the motor expression domain to reinforce children self-efficacy in that area.
**Objectives**	- To promote the internalization of self-regulatory questions associated to the forethought phase;<br>- To support the prosocial dimension of the learning process;<br>- To explore children self-efficacy perception on gross motor activities;<br>- To explore the outdoor places (if it is possible).
**Description of the activity**	Children pretend to cross the Cold River.<br>Teacher can prepare the crossing on a higher platform (i.e., on a bench or chairs) and on a lower platform (i.e., pieces that resemble stones or a narrow path).<br>Teacher should present the activity according to the self-regulatory principles and using the cards with the planning questions (i.e., explains the instruction; recalls the objective; anticipates the strategies and the resources needed; identifies children self-efficacy beliefs, interest in the activity and perception of difficulty).
**Degrees of freedom**	Children choose if they want to cross the river with or without help (i.e., holding hands with another child or adult). They can also decide if they want to cross the river in the high or low platform.
**Self-regulatory dimensions, processes and strategies to promote with this activity**	This activity allows to promote intentionally the emotional/behavioral dimension of the learning process.<br>Self-efficacy and self-reaction processes should also be improved. It is a suitable opportunity to promote adaptation/defense inferences and self-consequence strategies.<br><br>Self-regulatory questions to develop with children:<br>- How do you feel doing this activity? (emotional/behavioral dimension)<br><br>- Do you feel able to cross the Cold River? Why? *If you do it, what reward do you think you deserve?* (self-efficacy and self-consequence)<br><br>- In the next time, how are you going to do this activity? *Did you enjoy the activity? Why?* (self-reaction and adaptation/defense inferences)
**Suggestions**	Carry out the activity in an outdoor place. Use other available materials that resemble the river and nature.
**Materials**	Narrow and long bench or chairs; irregular pieces to support the feet.
