# Supplementary material for: Fostering self-regulated learning in preschool through dynamic assessment methodologies
Source: PLoS One. 2024 Mar 21;19(3):e0298759. doi: 10.1371/journal.pone.0298759 (PMC10956879; doi:10.1371/journal.pone.0298759)
Supplement: S2 File — (PDF) [file pone.0298759.s002.pdf]

Supporting information 2. Professional training contents

Phase	Contents
**Introduction/ modeling**	- Theoretical and conceptual approach about self-regulated learning as a cyclical and multidimensional process.<br>- Analysis of the self-regulated learning specificities in preschool in terms of age development.<br>- Reflection on the needs of assessing and promoting self-regulated learning in preschool.<br>- Presentation of resources and instruments to assess and promote self-regulated learning.<br>- Detailed analysis of the Dynamic Assessment of Self-regulated learning in Preschool method.
**Preparing and planning the educational intervention**	- Development of focus group interview with teachers about the first application of the Dynamic Assessment of Self-regulated learning in Preschool method with children.<br>- Approach to the three phases of the self-regulated learning cycle (i.e., forethought, performance and self-reflection), aiming to promote de use of self-regulated learning strategies with children; analysis of each phase specificities and integration of the cycle as a whole – "integration phase."<br>- Presentation of the educational intervention procedures and resources to promote self-regulated learning in preschool.<br>- Return of theoretical assumptions about self-regulated learning dimensions, processes and strategies: reinforcement of metacognitive modeling.<br>- Exploration of the questioning potentialities to promote self-regulated learning; exercise training and practices.<br>- Preparation of the teachers work with children and clarification of doubts.
**Monitoring the intervention**	- Checking and monitoring the development of the educational intervention by the teachers with their children.<br>- Revision of the intervention procedures and resources.<br>- Clarification of doubts.
**Assessment and conclusion**	- Validation of the teachers experience on planning, monitoring and reflection practices along the training process.<br>- Development of focus group interview with teachers about the second application of the Dynamic Assessment of Self-regulated learning in Preschool method with children.<br>- Assessment and group reflection on the practical implication of the training sessions to preschool teachers' professional development and to the educational context.
